# Development of Three Different NK Cell Subpopulations during Immune Reconstitution after Pediatric Allogeneic Hematopoietic Stem Cell Transplantation: Prognostic Markers in GvHD and Viral Infections

**DOI:** 10.3389/fimmu.2017.00109

**Published:** 2017-02-10

**Authors:** Sabine Huenecke, Claudia Cappel, Ruth Esser, Verena Pfirrmann, Emilia Salzmann-Manrique, Sibille Betz, Eileen Keitl, Julia Banisharif-Dehkordi, Shahrzad Bakhtiar, Christoph Königs, Andrea Jarisch, Jan Soerensen, Evelyn Ullrich, Thomas Klingebiel, Peter Bader, Melanie Bremm

**Affiliations:** ^1^Clinic for Pediatric and Adolescent Medicine, University Hospital, Frankfurt, Germany; ^2^GMP Development Unit, Hannover Medical School, Institute of Cellular Therapeutics, Hannover, Germany; ^3^LOEWE Center for Cell and Gene Therapy, Goethe University, Frankfurt, Germany

**Keywords:** NK cells, immune reconstitution, CD56, CD16, allogeneic transplantation, children, reference values

## Abstract

Natural killer (NK) cells play an important role following allogeneic hematopoietic stem cell transplantation (HSCT) exerting graft-versus-leukemia/tumor effect and mediating pathogen-specific immunity. Although NK cells are the first donor-derived lymphocytes reconstituting post-HSCT, their distribution of CD56^++^CD16^−^ (CD56^bright^), CD56^++^CD16^+^ (CD56^intermediate=int^), and CD56^+^CD16^++^ (CD56^dim^) NK cells is explicitly divergent from healthy adults, but to some extent comparable to the NK cell development in early childhood. The proportion of CD56^bright^/CD56^int^/CD56^dim^ changed from 15/8/78% in early childhood to 6/4/90% in adults, respectively. Within this study, we first compared the NK cell reconstitution post-HSCT to reference values of NK cell subpopulations of healthy children. Afterward, we investigated the reconstitution of NK cell subpopulations post-HSCT in correlation to acute graft versus host disease (aGvHD) and chronic graft versus host disease (cGvHD) as well as to viral infections. Interestingly, after a HSCT follow-up phase of 12 months, the distribution of NK cell subpopulations largely matched the 50th percentile of the reference range for healthy individuals. Patients suffering from aGvHD and cGvHD showed a delayed reconstitution of NK cells. Remarkably, within the first 2 months post-HSCT, patients suffering from aGvHD had significantly lower levels of CD56^bright^ NK cells compared to patients without viral infection or without graft versus host disease (GvHD). Therefore, the amount of CD56^bright^ NK cells might serve as an early prognostic factor for GvHD development. Furthermore, a prolonged and elevated peak in CD56^int^ NK cells seemed to be characteristic for the chronification of GvHD. In context of viral infection, a slightly lower CD56 and CD16 receptor expression followed by a considerable reduction in the absolute CD56^dim^ NK cell numbers combined with reoccurrence of CD56^int^ NK cells was observed. Our results suggest that a precise analysis of the reconstitution of NK cell subpopulations post-HSCT might indicate the occurrence of undesired events post-HSCT such as severe aGvHD.

## Introduction

The reconstitution of natural killer (NK) cells following allogeneic hematopoietic stem cell transplantation (HSCT) plays an important role in the response against residual malignant cells and the control of viral infections (1, 2). Independent of the graft source, NK cells typically regenerate within the first month following HSCT (3). However, there is an overrepresentation of CD56^bright^CD16^neg^ NK cells in the early phase post-HSCT compared to healthy individuals (4, 5), where NK cells are composed of about 90% CD56^dim^CD16^++^ and 10% CD56^bright^CD16^−^ cells. Whereas CD56^dim^CD16^++^ NK cells mediate cytotoxicity and antibody-dependent cellular cytotoxicity, the CD56^bright^CD16^−^ subpopulation, which is mainly present in the early period post-HSCT, primarily secretes immunoregulatory cytokines. Presumably, the development from CD56^bright^ to CD56^dim^ NK cells corresponds to sequential steps of NK cell differentiation (6). In most patients, the ratio between CD56^bright^ and CD56^dim^ NK cells normalizes within the first 6 months post-HSCT influenced by the patient’s age and events following HSCT. A correlation between the reconstitution of NK cells and overall survival was described by few studies emphasizing their essential role in the defense of infections when T cell immunity is mainly absent (7, 8). Furthermore, NK cells were successfully applied as immunotherapy for patients with high-risk malignancies suffering from impending relapse following HSCT. In clinical studies, adoptively infused NK cells induced graft-versus-leukemia/tumor effect without concomitant severe graft versus host disease (GvHD) (9–11). In addition, recent studies ascribe GvHD reduction to NK cell function post-HSCT (12). In this work, we focused on the regeneration of CD56^bright^ and CD56^dim^ NK cells with a special regard on the population shifting between these subpopulations. We divided NK cells in three NK cell subpopulations. In the first step, we established reference values of CD56^++^CD16^−^ (CD56^bright^), CD56^++^CD16^+^ (CD56^int^), and CD56^+^CD16^++^ (CD56^dim^) NK cells of healthy children and adolescents (*n* = 174). These reference values were matched and compared to the NK cell reconstitution of patients, who did not suffer from any viral infection or GvHD and are still alive after allogenic stem cell transplantation. In the next step, we investigated the associations between the reconstitution of the three different NK cell subpopulations in regard to the occurrence of events such as acute graft versus host disease (aGvHD) or chronic graft versus host disease (cGvHD) and severe viral infections in the first year post-HSCT in contrast to a cohort of patients without severe events assumed as control group.

## Materials and Methods

### Reference Cohort of Children and Adolescents

In this cross-sectional monocentric study (approval ethic committee Ref. No. 139/09), 174 donors (64 females and 110 males) were included. Residual peripheral blood samples of hematologically healthy children aged 1 month to 18 years were analyzed (patients were hospitalized, e.g., for cleft lip and palate correction). Inclusion criteria involved no incidence of immunodeficiency or infection (defined as >2 severe infections/year, >8 infections/year, persistent fungal infections, post-vaccinal complications, no evidence of acute bleeding, negative for CRP and normal leukocytes, lymphocytes, and neutrophil granulocytes).

### Patients and Grafts

The reconstitution of NK cells and their subpopulation CD56^bright^, CD56^int^, and CD56^dim^ was analyzed in *n* = 74 patients (*n* = 25 females and *n* = 49 males) transplanted from 2010 to 2016 (see Table 1). Indications for HSCT were high-risk acute lymphoblastic leukemia (*n* = 51), acute myeloid leukemia (*n* = 12), and myelodysplastic syndrome (*n* = 11). Median age at HSCT was 10.4 years (range: 1.3–24.4 years). Grafts were received from matched family donors (MSD; *n* = 20), matched unrelated donors (MUD) (*n* = 39), and haploidentical mismatched family donors with <8/10 HLA matches (MMFD; *n* = 15). No significant differences in the occurrence of GvHD were available in the different donor groups (MSD: 6/20, MUD: 10/39, and MMFD: 3/15). A second or third transplant was administered to *n* = 9 and *n* = 2 patients, respectively, because of relapse after first or second HSCT. Stem cell sources consisted of (bone marrow; *n* = 48), unmanipulated PBSC (*n* = 11), and T cell-depleted PBSC (*n* = 15). Post-transplant aGvHD occurred in *n* = 31 patients with grades I (*n* = 9), II (*n* = 3), III (*n* = 16), and IV (*n* = 3), cGvHD in 22 patients. Severe viral infections occurred in 18 patients including primary infection or reactivation with cytomegalovirus (CMV) (*n* = 8), adenovirus (ADV) (*n* = 5), and Epstein–Barr virus (EBV) (*n* = 5).

**Table 1:** **Patient’s characteristics**.

Groups	All	aGvHD	cGvHD	Viral infection	Control group
**Patients**	74	19	22 (8)	18	23

**Diagnosis**					
ALL	51	13	12 (4)	13	17
AML	12	3	3 (2)	5	3
MDS	11	3	7 (2)	0	3

**Age at HSCT**					
Median (range)	10.4 (1.3–24.4)	8.9 (1.9–17.6)	9.4 (1.3–18.2)	10.3 (3.4–17.7)	12.1 (2.3–24.4)

**Sex**					
Male	49	11	15 (6)	13	16
Female	25	8	7 (2)	5	7

**Donor type**					
MSD	20	6	11 (4)	1	6
MUD	39	10	9 (2)	9	13
MMFD	15	3	2 (2)	8	4

**Stem cell source**					
BM	48	12	17	7	16
Unmanipulated PBSC	11	4	3	3	3
T cell depleted	15	3	2	8	4

**GvHD**					
aGVHD	31	19	20	0	0
Grade I/II/III/IV	9/3/16/3	0/0/16/3	9/3/7/1	0/0/0/0	0/0/0/0
cGvHD	22	8	22	0	0

**Viral infections**					
ADV/CMV/EBV	5/8/5	0/0/0	0/0/0	5/8/5	0/0/0
**Survival (%)**	87	74	96	78	100

**Follow-up (months)**					
Median (range)	35 (3–105)	33 (3–77)	43 (6–92)	28 (2–103)	48 (24–105)

*ALL, acute lymphoblastic leukemia; AML, acute myeloid leukemia; MDS, myelodysplastic syndrome; HSCT, hematopoietic stem cell transplantation; MSD, matched sibling donor; MUD, matched unrelated donor; MMFD, mismatched family donor; BM, bone marrow; PBSC, peripheral blood stem cells; GvHD, graft versus host disease; aGvHD, acute graft versus host disease; cGvHD, chronic graft versus host disease; ADV, adenovirus; CMV, cytomegalovirus; EBV, Epstein–Barr virus*.

### Study Design

This study was carried out in accordance with the Declaration of Helsinki and was approved by the Medical Ethics Committee of the Frankfurt University Hospital (Ref. No. #198/16). Peripheral blood was collected within the framework of a post-HSCT routine sampling for clinical follow-up from 2010 to 2016. In engrafted patients, samples were collected starting at day 15, within the first year monthly, within the second year three monthly, and afterward every 6 months until 36 months post-HSCT, respectively. In total, *n* = 925 measurements of *n* = 74 patients were included in this analysis.

Depending on the occurrence of unexpected events such as aGvHD (only grades III and IV, grades I and II excluded), cGvHD, and severe viral infections, the study group was partitioned. The patient group without aGvHD/cGvHD or any viral infections was chosen for comparison as control group.

Patients showing a distinct CD56^int^ population in a sufficient absolute amount post-HSCT were elected for more detailed analysis applying a 10-color flow cytometry.

### Assessment of NK Cells and Their Subpopulations

Natural killer cell subpopulations were analyzed on a FC500 flow-cytometer (Beckman Coulter, Krefeld, Germany) applying a five-color panel to estimate CD3^+^ T cells, CD19^+^ B cell, and CD3^−^CD56^+^ NK cells, including the differentiation into CD56^++^CD16^−^, CD56^++^CD16^+^, CD56^+^CD16^++^ NK cells, abbreviated as CD56^bright^, CD56^int^, and CD56^dim^ NK cells, respectively. Absolute cell numbers were estimated from peripheral blood samples in a dual platform lyse-no-wash procedure as described previously (13). In brief, a tube of 100 µl EDTA-peripheral blood was labeled with CD45/CD56/CD19/CD3 tetraCHROME (clones B3821F4A/N901/J3-119/UCHT1) multi-color mAb conjugated with FITC, phycoerythrin (PE), phycoerythrin texas red (ECD), and phycoerythrin-cyanine 5 (PC5). CD16 phycoerythrin-cyanine 7 (PC7, clone: 6607118) was additionally added. For the measurement of T cells including T helper and cytotoxic T cells, we applied the tetraCHROME multi-color reagent CD45/CD4/CD8/CD3 (clones B3821F4A/SFCI12T4D11/SFCI21Thy2D3/UCHT1) conjugated with FITC, PE, ECD, and PC5. All reagents were acquired from Beckman Coulter Immutech (Marseille, France).

Differences in the expression profiles of CD56^bright^, CD56^int^, and CD56^dim^ cells were analyzed applying two panels on a Navios™ 10-color flow cytometer (Beckman Coulter, Krefeld, Germany). Panel 1: CD226 = DNAM-1 (FITC; clone: KRA236), NKG2A (PE; clone: Z199), DUMP = CD3&CD14&CD19 (ECD; clones: UCHT1, RMO52, J3-119), CD117 (PC5.5; clone: 104D2D1), CD27 (PC7; clone: 1A4CD27), CD56 [allophycocyanin (APC); clone: N901], CD127 (APC-A700; clone: R34.34), CD16 (APC-A750; clone: 3G8), CD57 [Pacific Blue (PB); clone: NC1], CD45 [Krome Orange (KrO); clone: J.33]. Panel 2: CCR5 (FITC; clone: 2D7), killer cell immunoglobulin-like receptor (KIR) mix (CD158 + CD158b + CD158e1; clones: EB6B, GL183, Z27.3.7), DUMP = CD3 + CD14 + CD19 (ECD; clones: UCHT1, RMO52, J3-119), CD117 (PC5.5; clone: 104D2D1), CX3CR1 (PC7; clone: 2A9-1), CD56 (APC; clone: N901), CD62L (APC-A700; clone: DREG56), CD16 (APC-A750; clone: 3G8), CCR7 (PB; clone: G043H7), and CD45 (KrO; clone: J.33). All antibodies were purchased from Beckman Coulter Immutech except CCR5 (BD Biosciences, Heidelberg, Germany) and CX3CR1 (Biolegend, San Diego, CA, USA). Staining was performed, using 100 µl of peripheral blood for each tube followed by 15 min of incubation at room temperature and erythrocyte lysis applying NH_4_Cl reagent (Beckman Coulter, Marseille, France).

Stained Cyto-Comp™ cells were applied to compensate the fluorescence overlap. The flow cytometer fluidic stability and the optical alignment were daily tested using Flow-Check™ Fluorospheres (Beckman Coulter, Krefeld, Germany). For verification, Immunotrol cells (Beckman Coulter) were applied three times a day. Furthermore, we participate in an external quality assessment for the detection of T-, B-, and NK cells (INSTANT e. V.—provider for German round robin test, No 213).

Data evaluation was performed using Kaluza and CXP-software (Beckman Coulter).

### Statistical Analysis

Statistical analysis was performed using GraphPad Prism 6 (GraphPad Software, San Diego, CA, USA). The NK subpopulation reference values of healthy children and adolescents were calculated with non-linear exponential regression analysis (equation: one-phase decay, least square fit; function: *Y* = (*Y*0 – Plateau) × exp(– *K* × *X*) + Plateau). Significant differences between groups were assessed by a non-paired two-tailed Mann–Whitney *U* test. *p*-Values <0.05 were regarded as significant and are indicated as *p < 0.05, **p < 0.01, ***p < 0.001, and ****p < 0.0001.

## Results

### Normalization of CD56^bright^ to CD56^dim^ Ratio Post-HSCT within the First Year Post-HSCT

**Figure 1:**
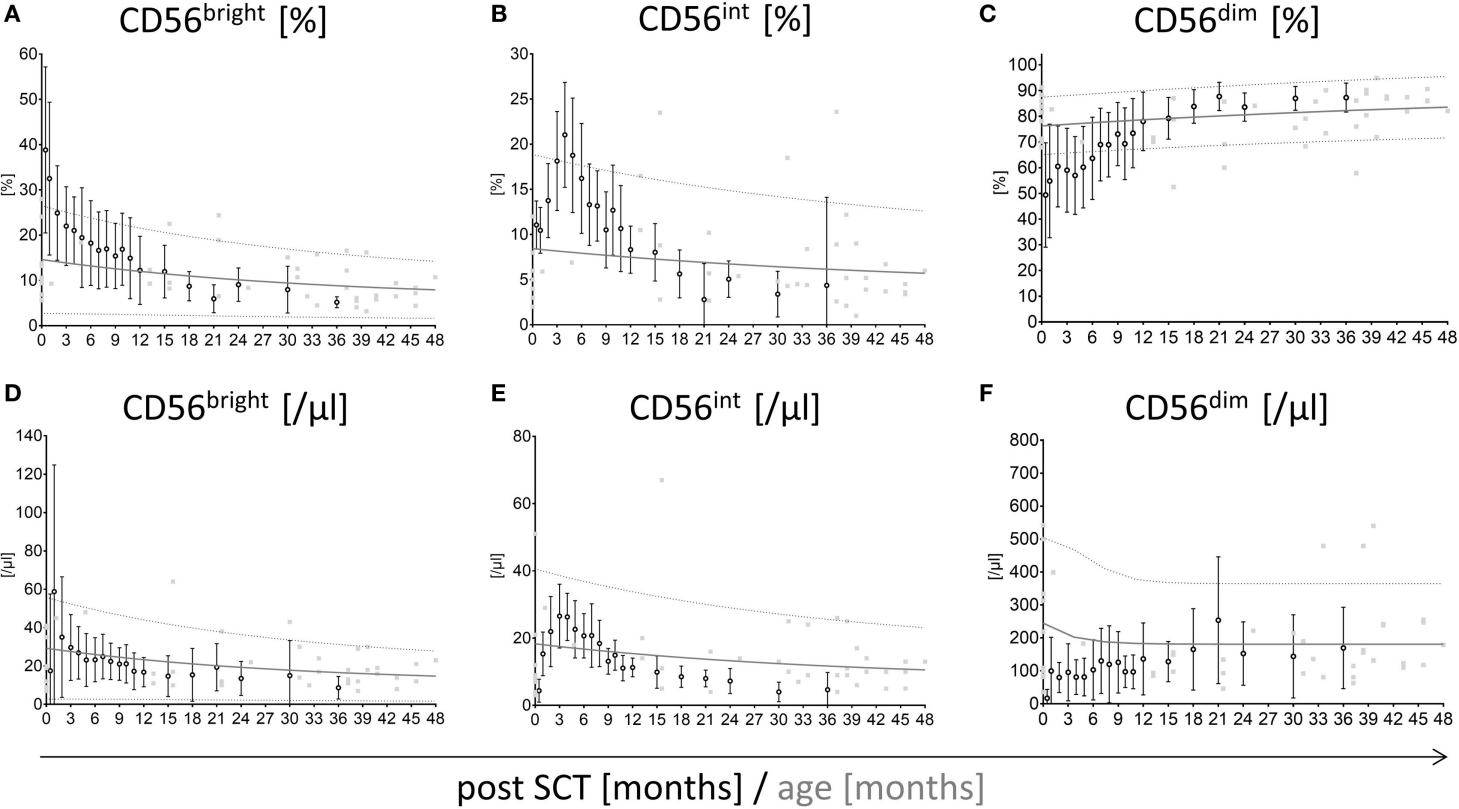
**Reconstitution of CD56^bright^, CD56^int^, and CD56^dim^ natural killer (NK) cells with respect to reference percentiles of healthy children**. Reconstitution of CD56^bright^, CD56^int^, and CD56^dim^ NK cells plotted into a graph showing the 10th, 90th (^….^, dotted line), and 50th (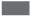, solid line) percentile of NK cell reference values of healthy children (*n* = 174) as well as their underlying measurements (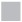, gray squares). Measurements of stem cell transplanted patients without graft-versus-host disease or viral infections (*n* = 23; mean with SD) were plotted into the reference model (**○**, black rimmed circles) at 19 time points until 36 months after hematopoietic stem cell transplantation (HSCT). Time points were 15 days, 1, 2, 3, 4, 5, 6, 7, 8, 9, 10, 11, 12, 15, 18, 21, 14, 30, and 36 months after SCT. The CD56^bright^ and CD56^dim^ NK cells post-HSCT needed around 12 months to reach the distribution of healthy individuals NK cells. At that time point, the CD56^bright^, CD56^int^, and CD56^dim^ NK cells met the 50th percentile of the reference range **(A–C)**. CD56^bright^ NK cells were elevated post-HSCT but already reached the reference range after 2 months **(A)**. Intermediate NK cells matched the reference range directly after HSCT but showed a fast proliferation until 5 months followed by a decline until 12 months post-HSCT **(B)**. CD56^dim^ NK cells were underrepresented directly after HSCT, reaching the lower reference range after 8 months post-HSCT **(C)**. The development of absolute cell counts of CD56^bright^, CD56^int^, and CD56^dim^ NK cells is shown in subfigures **(D–F)**.

The distribution of NK cell subpopulations post-HSCT is divergent from healthy individuals showing a high proportion of CD56^bright^ cells shifting toward CD56^dim^ NK cells. To estimate the reconstitution time to a normal CD56^bright^, CD56^int^, and CD56^dim^ NK cell distribution and absolute cell counts post-HSCT, we first generated age-matched reference values of NK cell subpopulation frequencies and absolute NK cell counts for healthy children and adolescents (Figure S1 in Supplementary Material). Interestingly, the proportion of CD56^bright^, CD56^int^, and CD56^dim^ in early childhood changed from 15, 8, and 78 to 6, 4, and 90% in adults, respectively. Subsequently, we matched the NK cell reconstitution of all patients of the control group without severe events (e.g., GvHD, infections) with the newly generated reference values (Figure 1). The frequency of CD56^bright^ NK cells is elevated directly after HSCT but reaches the upper reference range following 2 months. However, the levels of CD56^bright^ appear slightly increased remaining between the 50th and 90th percentile until 12 months post-HSCT (Figure 1A). Surprisingly, the percentage of CD56^int^ cells matches the reference range within the first 2 months post-HSCT but increases starting from 3 months exceeding the 90th percentile of the reference range following 5 months post-HSCT. After this period, the CD56^int^ fraction declines and converges to the 50th percentile of the reference values 12 months after HSCT (Figure 1B). By contrast, the CD56^dim^ fraction remains below the 10th percentile until 8 months and reaches the 50th percentile of normal reference values after 12 months post-HSCT (Figure 1C). Comparable reconstitution profiles are also apparent for absolute values of CD56^bright^, CD56^int^, and CD56^dim^ NK cells. However, absolute values after HSCT seem to be lower than NK cells of healthy children (Figures 1D–F).

### Reconstitution of NK Cells with Regard to GvHD Development

**Figure 2:**
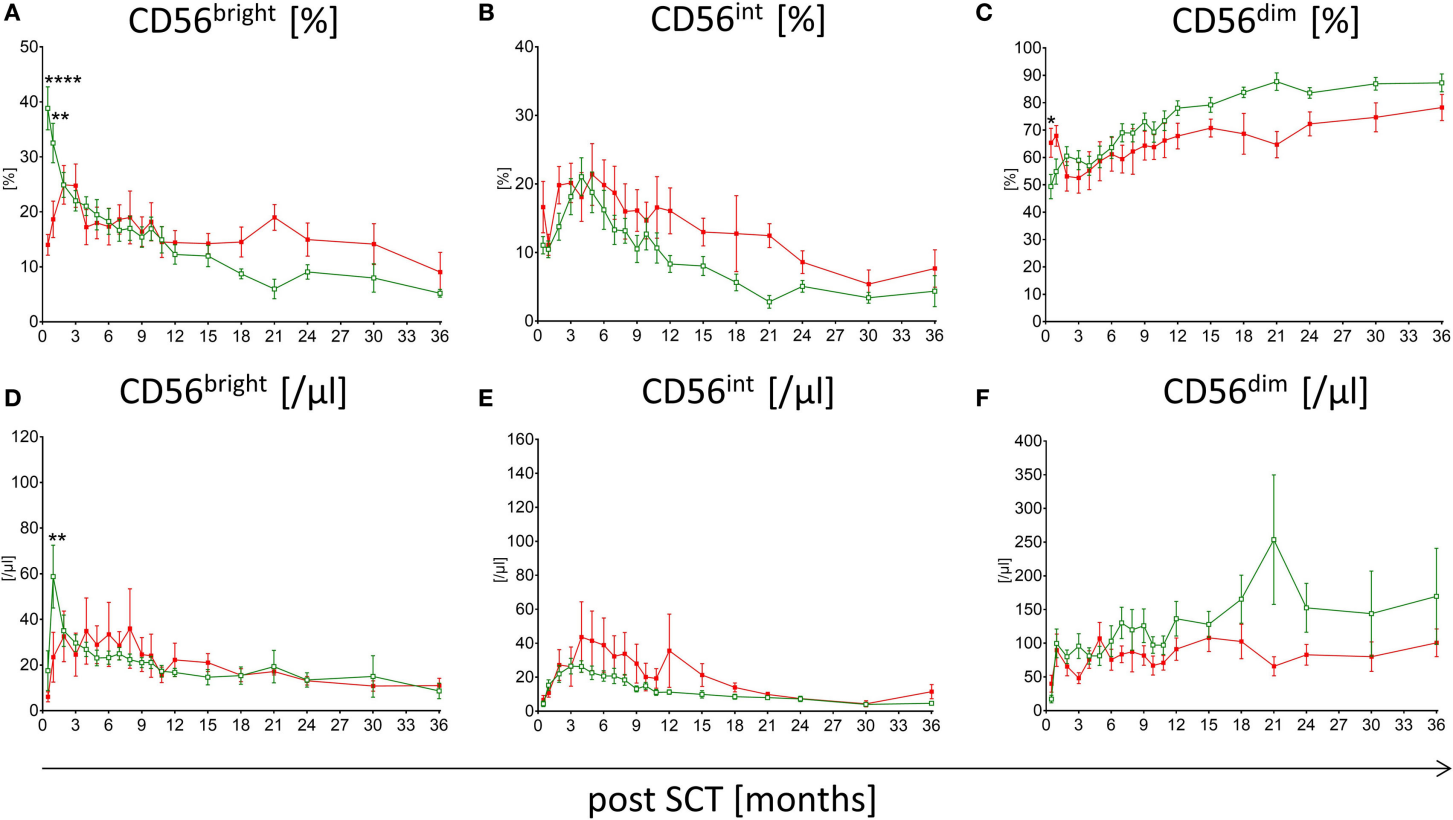
**Comparison of natural killer (NK) cell reconstitution in patients suffering from acute graft versus host disease (aGvHD) versus patients without events**. Regeneration of CD56^bright^, CD56^int^, and CD56^dim^ NK cells in patients affected with severe aGvHD with grade III and IV (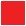, red squares, *n* = 19) and patients without events post-hematopoietic stem cell transplantation (HSCT) (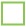, green rimmed squares, *n* = 23) at time point of 15 days, 1, 2, 3, 4, 5, 6, 7, 8, 9, 10, 11, 12, 15, 18, 21, 14, 30, and 36 months after SCT. Especially, in the first 3 months, post-HSCT aGvHD patients showed significantly lower CD56^bright^ NK cells in both percentage **(A)** (*p* < 0.0001) and absolute amounts **(D)** (*p* < 0.01). CD56^int^ NK cells of aGvHD patients seem delayed in their development as patients without events show an increased intermediate population over a period of at least 18 months **(B,E)**. Except in the beginning, CD56^dim^ NK cell levels of GvHD patients remain below those of patients without events during the whole monitoring interval **(C,F)**. Measurements were available at almost all time points, except for five patients of the aGvHD group who died 2, 3, 9, 9, and 18 months post-HSCT.

**Figure 3:**
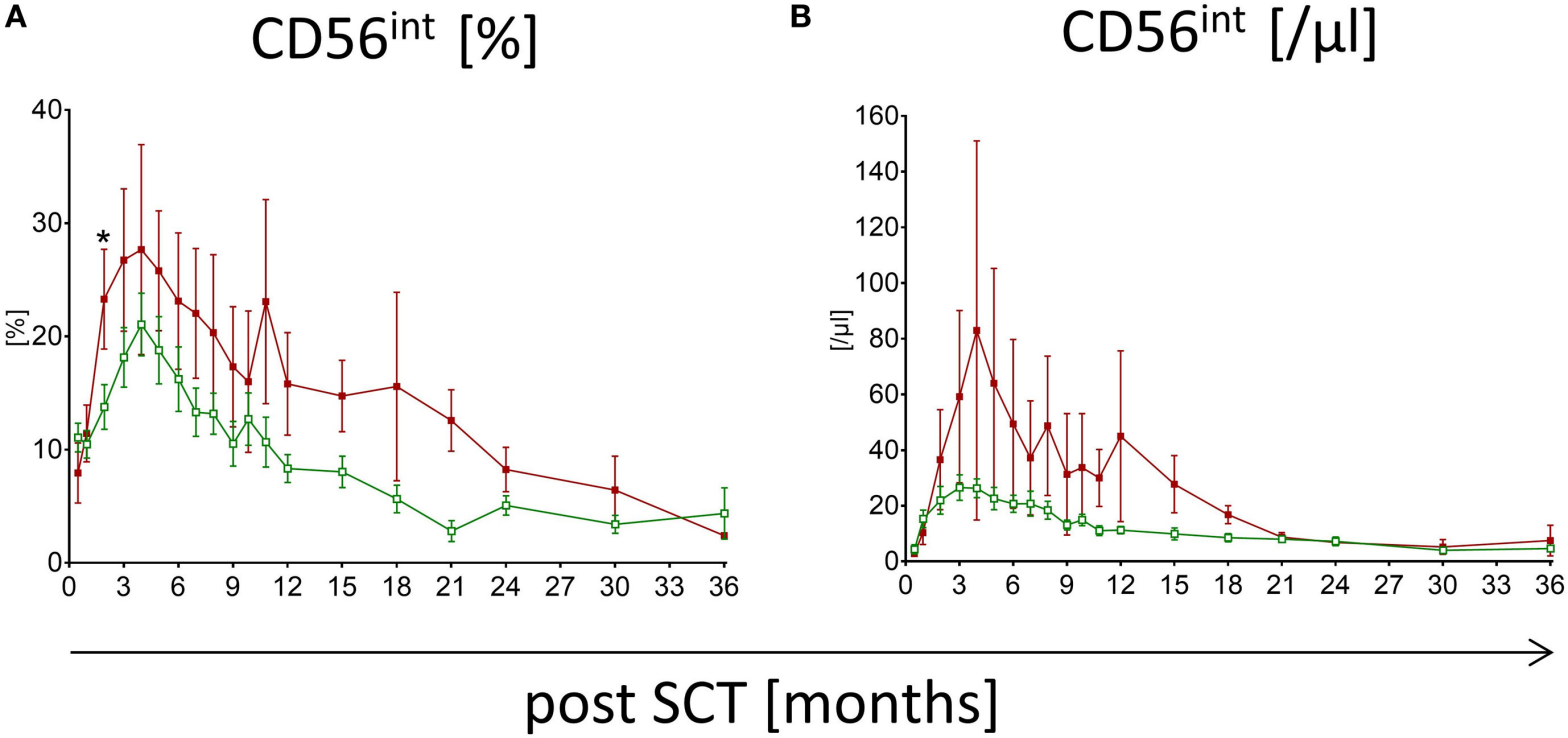
**Natural killer (NK) cell reconstitution in patients suffering from chronic graft versus host disease (cGvHD)**. Development of CD56^int^ NK cells of patients suffering from cGvHD after severe acute graft versus host disease (grade III or IV) is shown for frequency **(A)** and absolute count **(B)**. Over a period of at least 18 months, a higher amount of CD56^int^ NK cells for patients suffering from cGvHD (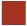, dark red squares, *n* = 8) was detectable compared to patients without severe events post-hematopoietic stem cell transplantation (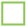, green rimmed squares, *n* = 23).

Patients suffering from aGvHD with grade higher than III show significant differences in the reconstitution of NK cell subpopulations compared to patients without any severe events post-HSCT. The median time of first symptoms of aGvHD was 22 days post-HSCT (range: 13–84). Within the first, second, and third month 68, 89, and 100% of the affected patients showed first signs of aGvHD, respectively. Patients without events show conspicuously higher frequency of CD56^bright^ NK cells within the first 3 months following HSCT compared to patients suffering from aGvHD with the most significant difference already 15 days following engraftment (*p* < 0.0001; Figure 2A). This could also be shown for the absolute CD56^bright^ NK cell amount 1 month after HSCT (*p* < 0.01; Figure 2D). This tendency was less pronounced when analyzing NK cells in patients with lower grades aGvHD (data not shown). Furthermore, the reconstitution of NK cell subpopulations seems to be delayed in patients suffering from aGvHD. For the CD56^int^ NK cell population, a displacement in time could be shown, which leads to a longer increase in CD56^int^ frequency and absolute amount (Figures 2B,E). To reach the 50th percentile of normal reference values, patients with aGvHD need a prolonged reconstitution time taking at least two times longer compared to patients without events. Patients with lower GvHD grades were lying in between (data not shown). Even after 3 years of monitoring, a trend toward higher frequency of CD56^bright^ and CD56^int^ NK cells concomitant with lower CD56^dim^ was seen in patients suffering from severe aGvHD (not significant, Figures 2A–C). These differences in the NK cell development post-HSCT were also found evaluating the absolute amounts of NK cell subpopulations (Figures 2D–F). Noteworthy, patients affected with aGvHD following HSCT also show a reduced absolute amount of CD56^dim^ NK cells after 3 years post-HSCT (Figure 2F). Analyzing absolute NK cell count (including all three subgroups), we did not see a correlation between patients with and without aGvHD (Figure S2A in Supplementary Material). Analyzing cytotoxic T cells, we detected that patients reaching levels of cytotoxic T cells above 1,500/μl within the first year post-HSCT developed in almost all cases an aGvHD (Figure S2B in Supplementary Material). But this fact was only true for less than 25% of the total aGvHD patient cohort. Furthermore, we evaluated the NK cell regeneration of patients with primary aGvHD grade III or IV that became chronic. Thereby, we detected important differences in the development of NK cell subpopulations from CD56^bright^ above CD56^int^ to CD56^dim^ NK cells. The development from CD56^int^ to CD56^dim^ NK cells is delayed for at least 2 years. Furthermore, patients with chronification of aGvHD with grade >III have a markedly elevated CD56^int^ frequency (Figure 3A), which is also clearly visible in absolute cell count of CD56^int^ NK cells (Figure 3B).

### Receptor Expression of CD56^bright^, CD56^int^, and CD56^dim^ NK Cells Post-HSCT

**Figure 4:**
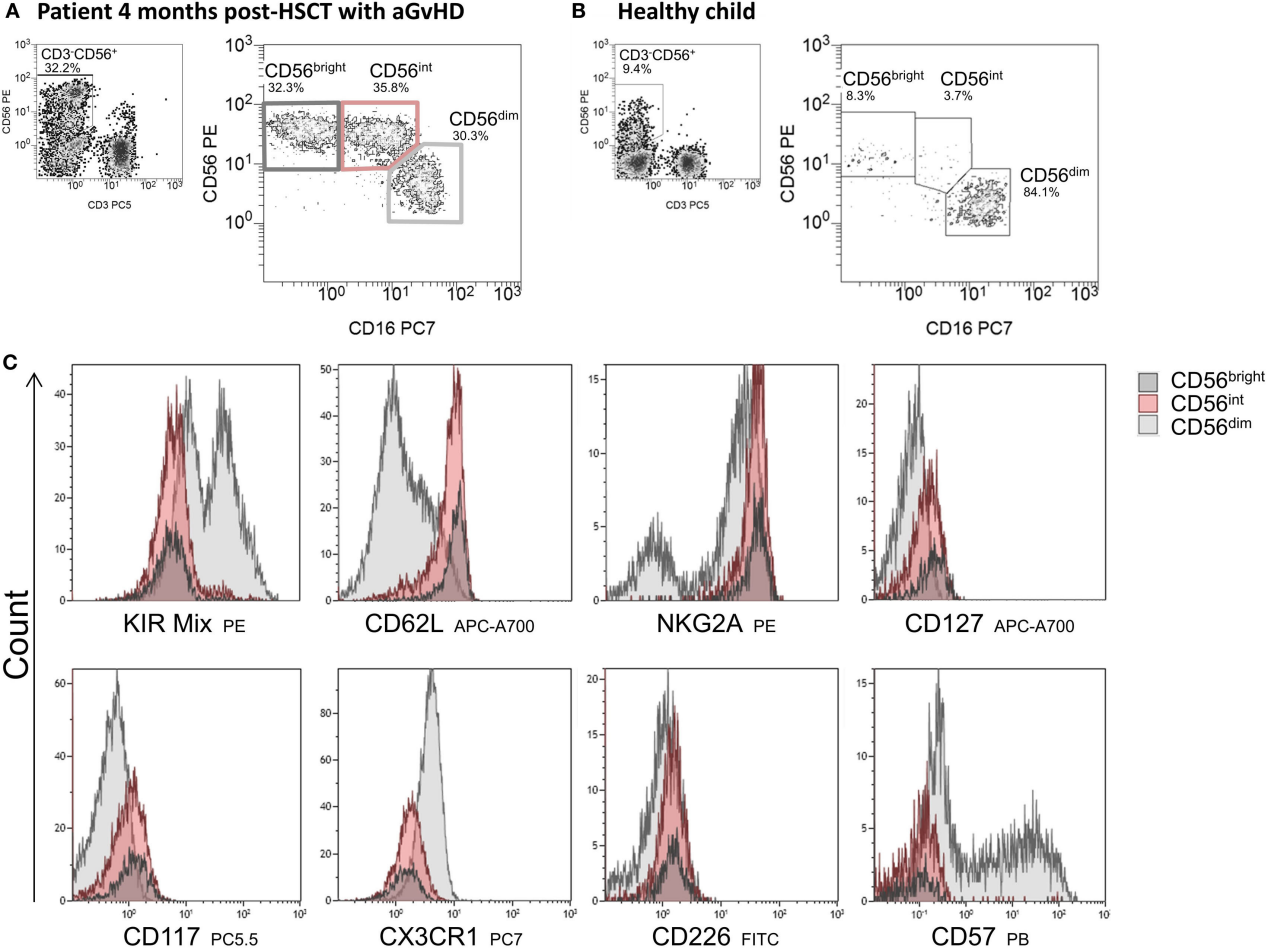
**Immunophenotyping of CD56^bright^, CD56^int^, and CD56^dim^ cells**. Exemplary flow cytometric plots of natural killer (NK) cell subpopulations of a patient 4 months post-hematopoietic stem cell transplantation (HSCT) with GvHD showing an almost equal distribution of CD56^bright^, CD56^int^, and CD56^dim^ cells **(A)** and of a healthy child aged 3 years with mainly CD56^dim^ cytotoxic NK cells **(B)**. NK cell subpopulations were characterized in detail by flow cytometric measurement of surface antigen expression of several ligands involved in adhesion, chemotaxis, and cytotoxicity. Expression of the corresponding receptors on NK cells is shown by overlay plots with MFI on the *x*-axis of respective antigen in allocation to CD56^bright^ (dark gray), CD56^int^ (red) compared to CD56^dim^ NK cells (light gray). CD56^bright^ and CD56^int^ NK cells show comparable receptor expression, but differ to CD56^dim^ cells in KIR-, CD62L, NKG2A, CX3CR1, and CD57 expression **(C)**.

As part of the patients had low absolute cell counts post-HSCT, a detailed phenotyping of the CD56^bright^, CD56^int^, and CD56^dim^ NK cell populations was only possible for an elected cohort of patients. To get an understanding of the function of CD56^int^ NK cells, we analyzed surface molecules linked to NK cell cytotoxicity, adhesion, and immune regulatory functions (e.g., chemokine and cytokine receptors) and compared the expression of KIRs, CD62L, NKG2A, CD127, CD117, CX3CR1, CD226, and CD57 on all three NK subpopulations. The CD56^dim^ population showed a higher expression of KIRs, whereas the CD56^int^ and CD56^bright^ population did not. However, not all KIRs applied within the mix were expressed with equal density (Figure 4). Regarding the homing receptor CD62L, the CD56^int^, and the CD56^bright^ fraction showed an increased expression compared to CD56^dim^ population. The expression of NKG2A was highest on CD56^int^ and CD56^bright^ cells but bipartite in CD56^dim^ NK cells. CX3CR1 is involved in adhesion and migration of NK cells and to a small extent higher presented on CD56^dim^ NK cells. As already described, we could also show that CD57 was only detectable on the CD56^dim^ subpopulation. Low expression of CD127 (IL7α chain), CD117 (c-Kit), and CD226 (DNAM-1) could be seen on all NK cell subpopulations; however, CD56^bright^ intend to have higher expression than CD56^dim^, whereas CD56^int^ was always lying in between. In summary, the expression profiles of CD56^int^ and CD56^bright^ NK cells were nearly congruent, but differed to CD56^dim^ cells in KIR, CD62L, NKG2A, CX3CR1, and CD57 expression (Figure 4).

### Influence of Viral Infection Post-HSCT on NK Cell Reconstitution

**Figure 5:**
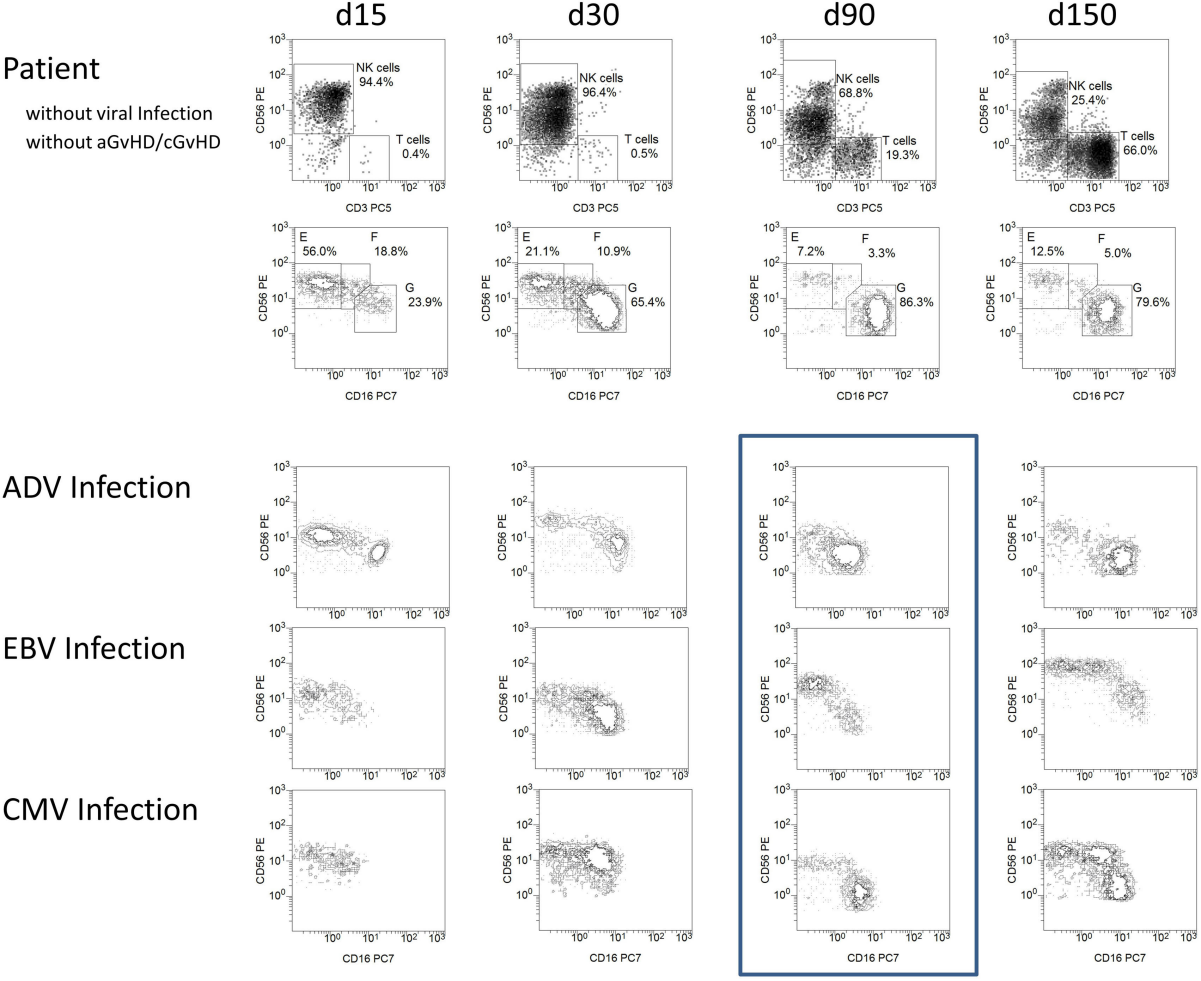
**Viral infections influencing natural killer (NK) cell reconstitution and NK marker expression density**. Exemplary flow cytometric plots of a patient without events and patients suffering from adenovirus (ADV), Epstein–Barr virus (EBV), and cytomegalovirus (CMV) infection between 15 and 150 days post-HSCT. Patients with high viral load (e.g., ADV above 40,000 genome equivalent/ml and CMV above 90,000 IU/ml) or qualitatively confirmed EBV infection with disease onset between day 30 and day 60 were exemplarily presented. NK cells of patients suffering from viral infection showed a slight reduction in CD56 and CD16 receptor expression 90 days post-HSCT (frame). After 150 days post-HSCT, the NK cells of the patient without events consisted of a major population of CD56^dim^, a small amount of CD56^bright^ and only few CD56^int^ cells whereas patients after EBV and CMV infection showed a distinct population of CD56^int^ NK cells again. Patients suffering from infections: ADV (*n* = 5), EBV (*n* = 5), CMV (*n* = 8).

The immune reconstitution of NK cell subpopulations post-HSCT was analyzed in patients without events and patients suffering from ADV (*n* = 5), EBV (*n* = 5), and CMV infection (*n* = 8). Infection was detected by the routine analysis of DNA copies in peripheral blood. Patients with elevated viral load at the day of transplantation were excluded from the study, resulting in a cohort of patients with occurrence of a positive viral load between 30 and 90 days post-HSCT. Interestingly, we observed a slight reduction in CD56 and CD16 expression in patients suffering from viral infection in between day 30 and day 60 post-HSCT measured by mean fluorescence intensity (Figure 5). After viral clearance in most patients, a considerable loss in absolute CD56^dim^ NK cell count occurred followed by continued regeneration of CD56^int^ NK cells, which was lower in patients without events post-HSCT on day 150 post-HSCT (Figure 5).

## Discussion

Especially in the early phase following allogeneic HSCT, together with neutrophils, NK cells are the first line of immune defense. Their immune reconstitution is of crucial importance for transplantation outcome with special regard to the occurrence of GvHD and viral infections. In this project, we analyzed NK cell subpopulations in detail not only focusing on CD56^bright^ and CD56^dim^ cells but also the fraction in between those subsets. With regard to NK cell subpopulation development in young healthy children, we found that it takes around 12 months until CD56^bright^, CD56^int^, and CD56^dim^ NK cells of patients post-HSCT reach the 50th percentile of age-matched reference range. Comparable results were published by Pical-Izard et al. describing that rapidly re-emerging NK cells remain immature for more than 6 months (8). Directly after HSCT we detected a highly increased frequency of CD56^bright^, whereas CD56^int^ NK cells correspond to the reference range, but considerably expand within the first 3 months post-HSCT. In contrast, CD56^dim^ NK cells deserve around 8 months to enter the reference range. These results confirm the hypothesis of sequential development of NK cells with CD56^int^ NK cells representing an intermediate state from CD56^bright^ to CD56^dim^ NK cells (14, 15). These CD56^bright^ NK cells in peripheral blood are closely related to those NK cells populating secondary lymphoid tissues (16). Further evidence supporting this hypothesis was published by Freud et al. describing that the CD56^bright^ subset is the major NK cell population that is derived early *in vitro* when CD34^+^ HPC are cultured in NK development supportive conditions, whereas CD56^dim^ NK cells develop later (17). Furthermore, CD56^bright^ NK cells display longer telomeres than the CD56^dim^ NK cells, indicating lower proliferation capacity (6).

We further characterized all three NK cell subpopulations with the finding, that CD56^int^ presented antigen expressions among CD56^bright^ and CD56^dim^ NK cells, even so CD56^bright^ and CD56^int^ NK cells showed rather equal expression profiles and seemed related more to CD56^bright^. However, differential expression of KIRs, CD62L, NKG2A, and CD57 was observed on CD56^dim^ NK cells. This is in parallel to other findings describing an increased expression of NKG2A, the IL-7 receptor (CD127) and the lymph node homing receptor CCR7 on CD56^bright^ cells (2, 5, 8, 18, 19) whereas CD56^dim^ NK cells acquire KIR, NKG2C, and CD57 expression (20).

Promoted by the IL-15 rich cytokine milieu post-transplant, NK cells are known to be one of the first lymphocyte subpopulation recovering post-HSCT (21). Therefore, NK cell reconstitution might be the basis for generating early prognostic markers regarding the occurrence of severe events and transplantation outcome. Kim et al. published that NK cell counts after allo-HSCT, especially on day 30, were predictive markers for GvHD, non-relapse mortality, and survival (22). Furthermore, there is evidence that the speed of NK cell reconstitution correlates with transplant outcome, suggesting their important role in the early period when specific T cell immunity is absent (7, 8). Our and other findings suggest that the monitoring of NK cell subsets in the early phase post-HSCT might provide first signs of aGvHD development (23). Interestingly, within the first 2 months post-HSCT patients without aGvHD or viral infections had significantly elevated levels of CD56^bright^ NK cells compared to patients suffering from aGvHD. This might be an early prognostic factor regarding GvHD development; however, it needs to be confirmed in a prospective study. Likewise results were also published by Kheav et al. showing an impaired reconstitution of CD56^dim^ NK cells 3 months post-HSCT (24). We also found a comparable trend for NK cell regeneration in patients suffering from cGvHD, although not significant (data not shown). This might be explained by the fact, that for aGvHD analysis, only patients suffering from GvHD grades III and IV were considered, whereas no differentiation was available regarding cGvHD (e.g., chronification of primary aGvHD grades I and II).

Literature is discordant whether steroids/immunosuppression have a negative impact on NK cell reconstitution. Giebel et al. proposed that the use of steroids for GvHD prophylaxis negatively affects quantitative reconstitution of NK cells after allo-HSCT (25). Although, patients suffering from GvHD grades III and IV normally receive steroids in our transplantation unit, we did not see any significant differences in the quantitative reconstitution of absolute NK cell numbers. Interestingly, Wang et al. described that CSA suppresses the *in vitro* proliferation of NK cells, especially the CD56^dim^CD16^+^KIR^+^ NK cells, resulting in a relative increase in the number of immature CD56^bright^CD16^−^KIR^−^ NK cells (26). This might also contribute to the delayed NK cell development in patients suffering from higher grade aGvHD that we observed within this study. However, this remains controversial as other studies analyzed the effect of CSA on NK cell function in short-term cultures and their cytokine production without finding significant differences between NK cells with and without CSA treatment (27, 28).

In patients suffering from viral infection post-HSCT, we observed a slight reduction in CD56 and CD16 expression. Notably, other publications already described the existence of CD56^−^CD16^+^ NK cells (CD56^negative^) NK cells in viral infections (e.g., HIV, hepatitis C), where NK cells undergo numerous phenotypic and functional changes (29). This CD56^negative^ subset has been associated with high HIV viral load and has been reported to have an impaired cytolytic function and cytokine production (30). This increase occurred primarily at the expense of CD56^dim^ NK cells, whereas numbers of CD56^bright^ NK cells remained stable (31). Furthermore, we observed a considerable loss in absolute CD56^dim^ NK cells followed by continued regeneration of CD56^int^ NK cells. Alteration of NK cells upon viral infection has already been shown by other research groups, for example Pical-Izard et al. showed that in patients being affected by CMV reactivation, NK cells showed lower degranulation and TNF-α production compared to patients without CMV reactivation post-HSCT (8). In addition, it was shown that CMV reactivation is followed by an increase in the proportion of NKG2C^+^ NK cells within 2–4 weeks, which persist for at least a year (32, 33).

In conclusion, only after around 12 months, NK cells post-HSCT reconstitute to a distribution of the subpopulations CD56^bright^, CD56^int^, and CD56^dim^ comparable to age-matched healthy controls. The expression profiles of CD56^int^ and CD56^bright^ NK cells resemble each other but differed in KIR, CD62L, NKG2A, CX3CR1, and CD57 expression to CD56^dim^ NK cells. We observed elevated levels of CD56^bright^ directly after and CD56^int^ NK cells 3 months post-HSCT accompanied by reduced CD56^dim^ NK cells supporting the hypothesis of sequential NK cell development. Furthermore, we analyzed alterations in NK cell development in patients with severe viral infections and GvHD. Following viral infection, there was a slight reduction in CD56 and CD16 receptor expression followed by a considerable loss in absolute CD56^dim^ NK cells and continued regeneration of CD56^int^ NK cells. Most important, within the first 2 months, post-HSCT patients without severe events had significantly elevated levels of CD56^bright^ NK cells compared to patients suffering from aGvHD. While first measurements performed as early as 15 days following HSCT revealed the most significant differences, clinical occurrence of aGvHD was observed in median on day 22 post-HSCT. Therefore, we recommend immunophenotyping of NK cell subpopulations directly following engraftment, which might be an early prognostic factor regarding GvHD development.

## Author Contributions

Study design: MB, SH, RE, and EK. Performed the experiments: JB-D and SBe. Provided clinical data: JS, AJ, SBa, and PB. Analyzed the data: SH and EK. Coordinated the research: SH and CC. Contributed reagents/materials/analysis tools: MB, SH, VP, JB-D, and SBe. Performed statistical analyses: SH and ES-M. Wrote the manuscript: MB and SH. Discussed data and revised the manuscript: CC, PB, VP, EU, and CK. Supervised the research: PB and TK. All the authors read and approved the final manuscript.

## Conflict of Interest Statement

The authors declare that the research was conducted in the absence of any commercial or financial relationships that could be construed as a potential conflict of interest.
