# Supplementary figures and images for: Development of Three Different NK Cell Subpopulations during Immune Reconstitution after Pediatric Allogeneic Hematopoietic Stem Cell Transplantation: Prognostic Markers in GvHD and Viral Infections
Source: Front Immunol. 2017 Feb 10;8:109. doi: 10.3389/fimmu.2017.00109 (PMC5300968; doi:10.3389/fimmu.2017.00109)

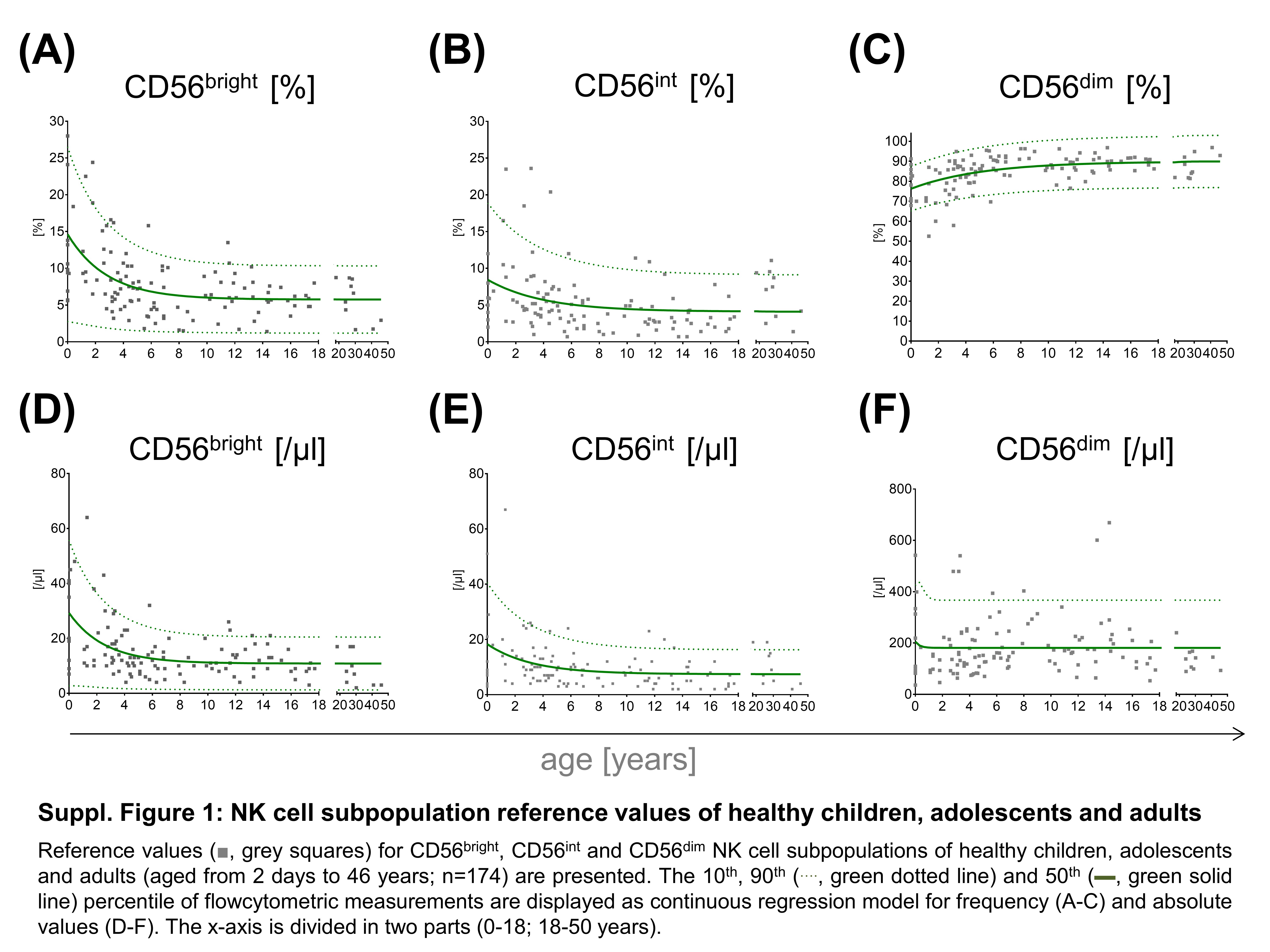

Supplement: Supplementary file 1 [file image_1.jpeg]

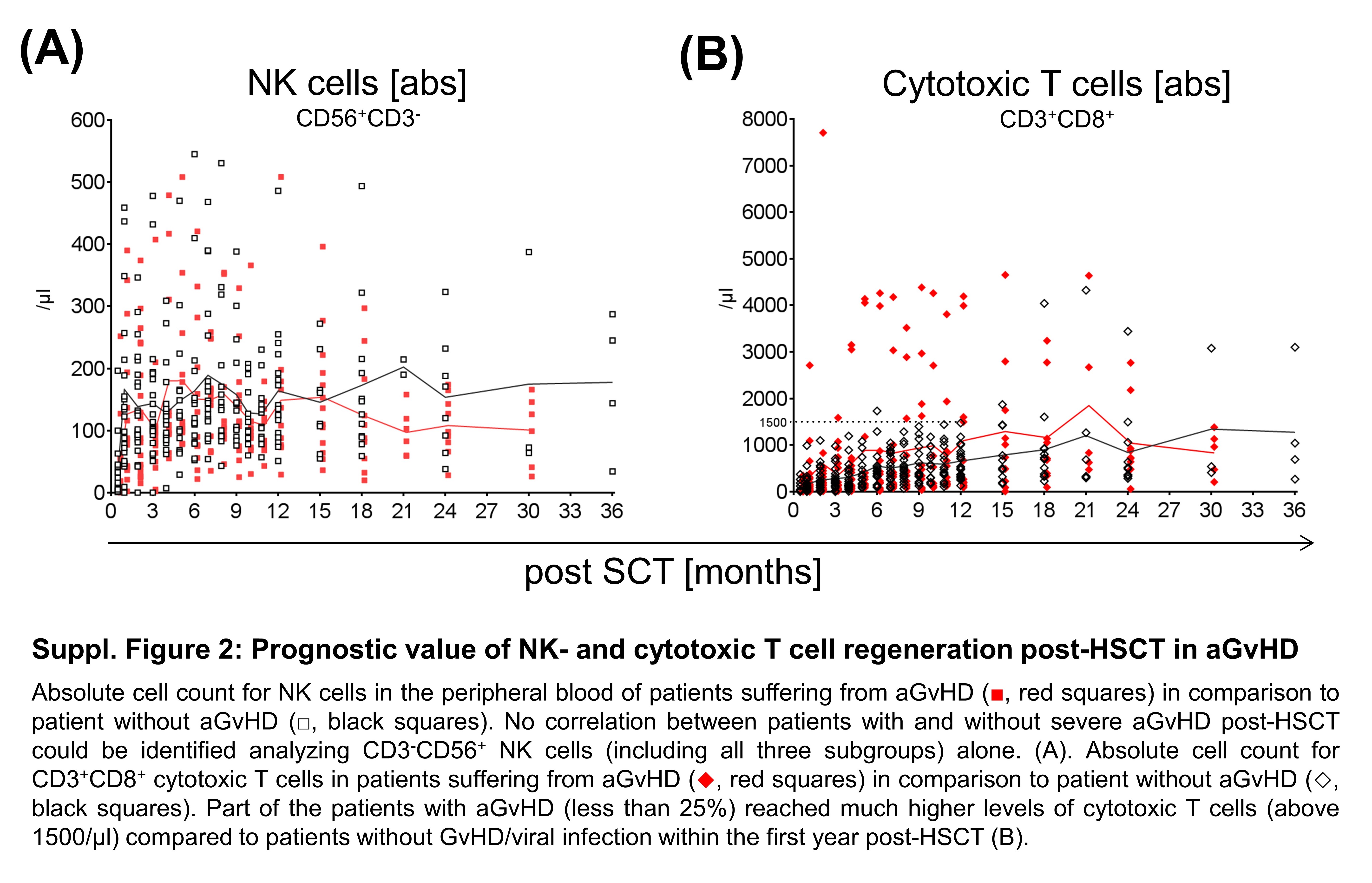

Supplement: Supplementary file 2 [file image_2.jpeg]
